# Quantitative screening of the pharmaceutical ingredient for the rapid identification of substandard and falsified medicines using reflectance infrared spectroscopy

**DOI:** 10.1371/journal.pone.0202059

**Published:** 2018-08-10

**Authors:** Graham Lawson, John Ogwu, Sangeeta Tanna

**Affiliations:** Leicester School of Pharmacy, Faculty of Health and Life Sciences, De Montfort University, Leicester, United Kingdom; Aligarh Muslim University, INDIA

## Abstract

The World Health Organization suggests that approximately 10% of medicines worldwide are either falsified or substandard with higher figures in low and middle income countries. Such poor quality medicines can seriously harm patients and pose a threat to the economy worldwide. This study investigates attenuated total reflectance-fourier transform infrared (ATR-FTIR) spectroscopy as a simple and rapid method for determination of drug content in tablet dosage forms. Paracetamol was used as the model pharmaceutical ingredient. Spectra of standard mixtures of paracetamol with different excipients formed the basis for multivariate PLS based quantitative analysis of simulated tablet content using different selected infrared absorbance bands. Calibration methods using ATR-FTIR were compared with the ATR-FTIR and conventional ultraviolet spectroscopic analyses of real tablet samples and showed that the paracetamol/microcrystalline cellulose mixtures gave optimum results for all spectral bands tested. The quantitative data for band 1524–1493cm^-1^ was linear (R^2^ ˃ 0.98; LOQ ≥ 10%w/w tablet). Global examples of paracetamol tablets were tested using this protocol and 12% of the tablet samples examined was identified as substandard. Each sample analysis was completed in just a few minutes. ATR-FTIR can therefore be used in the rapid screening of tablet formulations. The simplicity of the proposed method makes it appropriate for use in low and middle income countries where analytical facilities are not available.

## Introduction

The growing problem of substandard and falsified pharmaceuticals presents a serious and increasing threat to international public health and patient safety [1–3]. Substandard medicines result from poor manufacturing and quality assurance processes and reach the public due to lax control measures whereas falsified (counterfeit) medicines are deliberately and fraudulently labelled [4]. In reality, both falsified and substandard medicines claim to be something that they are not. The World Health Organization (WHO) has recently introduced the terms ‘substandard and falsified (SF) medical products’ [5] in an attempt to change the situation where there is no globally accepted definition for such medicines and poorly coordinated legal controls that seem to encourage the production of poor quality medicines [6]. The WHO estimated that about 10% of the global supply of medicines were falsified or substandard [7, 8]. In countries where there is good control of the supply of medicines the incidence of substandard or falsified medicines is reported to be around 1% whilst in low and middle income countries (LMIC) the level of substandard or falsified medicines rises to 50% of all medicines [9]. Up to 50% of medicines purchased from the internet may be of a poor quality [10], although a recent review has highlighted that it is difficult or impossible to make reliable estimations about the prevalence of falsified and substandard medicines [11]. These medicines can pose a significant threat to public health and produce economic problems worldwide and there is a need for improvements in methods for both screening and monitoring medicines for such poor quality medicines.

Examples of falsification of medicines include lifesaving anticancer, HIV and cardiovascular therapy medicines as well as lifestyle drugs such as weight loss/slimming pills and dietary supplements [7, 12, 13]. High demand over-the-counter (OTC) medicines for mild pain relief and antipyretics to reduce fever have also been the target for falsification. Paracetamol, also known as acetaminophen (4-acetamidophenol, *N*-acetyl-*p*-phenacetin), [14] has been identified as the second most commonly used active pharmaceutical ingredient (API) after acetylsalicylic acid and paracetamol containing OTC medicines have therefore been targeted by counterfeiters [15]. Paracetamol has also been used, in place of the specified API, in falsified medicines [16]. Reports of a situation in the USA where 500mg paracetamol tablets were actually labelled as 325mg suggest that people in both industrialised and LMICs are exposed to miss-labelled medication [17].

Historically, analytical techniques for determining the authenticity of medications have been based on the determination of the API content [13, 18, 19]. These pharmacopoeia approved methods [20–22] require sample preparation which includes solvent extraction of the APIs followed by filtration and/or dilution prior to analysis [2, 23]. Techniques such as high-performance liquid chromatography (HPLC) with ultraviolet (UV) detection [24], liquid chromatography coupled with mass spectrometry (LC-MS) [25] and nuclear magnetic resonance (NMR) spectroscopy [26] and have been used in the authentication of various tablet formulations. The chromatography-based techniques require large volumes of expensive solvents and have long analysis times due to complex sample preparation steps which make them unsuitable for rapid, simple and cheap analysis. These procedures also require well trained staff and well equipped laboratories [6, 27] which are not readily available in LMICs. With the growing prevalence of falsified and substandard medicines globally, the provision of simple, fast and affordable methods of analysis for screening these medications would be beneficial especially in LMIC [27–29].

Vibrational spectroscopic techniques are widely documented as being suitable analytical methods for the authentication of pharmaceutical products [30–34]. There has also been increased interest in the use of handheld devices that use spectroscopic analyses for screening medicines in the field [6, 18, 35]. Raman spectroscopy, in particular, has been applied extensively in the characterization and identification of suspected medicines [34, 36, 37] and also in the quantification of paracetamol [38]. Furthermore, near infrared spectroscopy (NIR) has been applied in this regard and in combination with Raman spectroscopy for authentication of falsified medicines [31, 39]. There is also the issue of chemical peaks not being well defined or separated (poor chemical peak specificity) in NIR spectroscopy making spectra difficult to interpret [35, 40].

Most applications of fourier-transform infrared (FTIR) spectroscopy for pharmaceutical analysis, like other spectroscopic techniques, include characterisation and identification of the presence or absence of active pharmaceutical ingredients (APIs) and excipients [31, 32]. Conventional FTIR with multivariate analysis has also been used for the quantification of APIs in antidiabetic drugs [41] and paracetamol [42] in solid pharmaceutical dosage forms. This approach requires skilled personnel since samples must be prepared as crushed powders, then finely dispersed in a KBr matrix which is then compressed into discs before analysis. This approach is time consuming, requires a source of continually dried KBr and a hydraulic press to produce uniform KBr discs.

Attenuated total reflectance fourier transform infrared spectroscopy (ATR-FTIR) has revolutionized conventional FTIR by eliminating the main challenges in the analysis of pharmaceutical solid dosage forms notably the time spent in sample preparation which involves sample extraction or KBr disc preparation and also the lack of spectral reproducibility. ATR-FTIR is quicker than some of the pharmacopoeia approved methods and conventional FTIR since the samples can be analysed directly to provide qualitative data or powdered before analysis [23, 42] if reproducible quantitative data is required.

The aim of this study was to develop and improve the simple, fast and cost effective method using ATR-FTIR to distinguish between genuine and falsified or substandard paracetamol tablets reported previously [23]. In the previous work the effect of different integration modes to manually determine areas of single characteristic peaks (univariate data) was combined with simple Beer Lambert Law calibration plots for quantitative measurements. The current work includes an assessment of the potential of the ATR-FTIR analysis of whole paracetamol tablets to confirm the presence of the API. A quantitative determination of the API level requiring the preparation of paracetamol-excipient powder calibration mixtures for ATR-FTIR analysis and a study of the effects of the use of different model excipients on the calibration results obtained using different spectral ranges will be conducted. Data will be processed by the in board automated multivariate calibration algorithm. This work will also include validation of the API levels determined by ATR-FTIR spectroscopy against a conventional UV spectroscopy quantitative analysis. The validated quantitative ATR-FTIR method will be applied to paracetamol tablet samples collected opportunistically from various countries globally.

These results aim to demonstrate the possible usefulness of the technique in low and middle income countries due to ease of sample preparation, the use of a green and low cost analytical method, rapid analysis and easy interpretation of the results.

## Materials and methods

### Reference chemicals and reagents

Analytical grade paracetamol was obtained from Sigma Aldrich, Dorset, UK. Excipients: maize starch, microcrystalline cellulose (MCC), magnesium stearate, and UV grade methanol were obtained from Fisher Scientific Ltd, Loughborough, UK.

### Test tablet samples

Tablet formulations containing paracetamol, for analysis, were obtained opportunistically, from outlets readily available to tourists or visitors in Europe, Asia, Africa and the Caribbean Islands. Table 1 summarizes the country of origin and expected dose of each of the tablets analysed.

**Table 1:** Paracetamol tablets analysed and their origin and expected amount.

Country (number of tablets)	Tablets analysed*		Paracetamol tablet labelled as	Expected amount (mg)
**UK (n = 2)**	UK P1T1	UK P1T2	Paracetamol	500
**Cyprus (n = 2)**	Cyp P1T1	Cyp P1T2	Remedol	
**Switzerland (n = 2)**	Swz P1T1	Swz P1T2	Dafalgan	
**Spain (n = 6)**	Spn P1T1	Spn P1T2	Panadol	
	Spn P2T1	Spn P2T2	Paracetamol Teva	650
	Spn P3T1	Spn P3T2	Paracetamol Teva	1000
**Belgium (n = 2)**	Bel P1T1	Bel P1T2	Paracetamol EG	
**India (n = 19)**	Ind P1T1	Ind P1T2	P1- Crocin Advance	500
	Ind P2T1	Ind P2T2	P2- G-mol	
	Ind P3T1	Ind P3T2	P3- Paracin	
	Ind P3T3	Ind P4T1	P4- Doliprane	
	Ind P4T2	Ind P5T1	P5- Calpol	
	Ind P5T2	Ind P5T3	P5- Calpol	
	Ind P6T1	Ind P6T2	P6- Crocin Advance	
	Ind P7T1	Ind P7T2	P7- Crocin Advance	
	Ind P8T1	Ind P8T2	Tharfenac	325
	Ind P9T1		Paracip	650
**Pakistan (n = 2)**	Pak P1T1	Pak P1T2	Panadol	500
**Nepal (n = 8)**	Nep P1T1	Nep P1T2	P1- Algina	
	Nep P2T1	Nep P2T2	P2- Algina	
	Nep P3T1	Nep P3T2	P3- Niko	
	Nep P4T1	Nep P4T2	P4- Algina	
**China (n = 4)**	Chn P1T1	Chn P1T2	P1- Tylenol	
	Chn P2T1	Chn P2T2	P2- Eurocetamol	
**UAE (n = 4)**	UAE P1T1	UAE P1T2	P1- Pmol	
	UAE P2T1	UAE P2T2	P2- Adol	
**Rwanda (n = 6)**	Rwa P1T1		P1- Ubithera	500
	Rwa P2T1		P2- Paradana	
	Rwa P3T1	Rwa P3T2	P3- Pharmaquick	
	Rwa P4T1	Rwa P4T2	P4- Eskay	
**Ghana (n = 4)**	Gha P1T1	Gha P1T2	P1- Ayrton	
	Gha P2T1	Gha P2T2	P2- Cetapol	
**Jamaica (n = 4)**	Jam P1T1	Jam P1T2	P1- Panadol	
	Jam P2T1	Jam P2T2	P2- Panadol	

Note: n = number of samples

*P1T1 = Pack 1 Tablet 1 and so on.

Tablet assays for content uniformity are usually based on the analysis of 20 tablets or 10 selected at random. In forensic scenarios there may not be this number of tablets available, the loss of 20 expensive antimalarial or HIV tablets is too wasteful for some countries and a single tablet assessment is more representative of the actual dose taken by a patient.

### Instrumentation

#### ATR-FTIR spectroscopy

All spectra were recorded on the Bruker Alpha FTIR spectrometer (Bruker Corporation, Germany) equipped with the ATR platinum diamond sampling stage to provide robustness and durability. Spectral acquisition was done using OPUS software version 7.5 (Bruker Corporation, UK).

For qualitative analyses the Spectrum Search facility was used. Quantitative measurements were carried out automatically using the QUANT2 facility within OPUS 7.5 to provide calibration spectra from different levels of paracetamol prepared in selected excipients.

#### UV-Vis spectrophotometry

UV spectra were collected using UV-Visible spectrophotometer, Helios Gamma (Thermo Electron Corporation, England). The spectral range considered was 190–400 nm with a scan interval of 0.5 nm. Quantitative readings were taken at a wavelength of 243.5nm. The UV-Visible spectrophotometer was controlled using the Vision Lite software 2.2 (Ueberlingen, Germany).

### Methods

#### Reference spectra

All spectra were measured in absorbance mode. A fresh background spectrum was measured against air before starting measurements and subsequently after every 5 runs. Small amounts of finely ground samples of the individual reference materials, paracetamol and excipients commonly used in pharmaceutical formulations, were placed on the diamond sampling crystal and pressed using a clamp to ensure proper contact. Each spectrum was measured by averaging 20 scans over the range 4000-400cm^-1^ with spectral resolution 2cm^-1^. Estimated scan time for spectral acquisition was 25 seconds. This process was repeated 5 times to ensure replicate data was produced. The platinum diamond sampling surface was cleaned after each sample using paper tissue with isopropanol and allowed to dry. Recorded fingerprint spectra for the reference materials were assessed for spectral reproducibility by comparing replicate spectra. Reproducible spectral data were used to create a local reference library. Members of the same excipient groups, starches, celluloses and stearates all had very similar spectra. Characteristic regions of the paracetamol spectrum were identified where there was little or no absorbance from excipient materials.

#### Reference paracetamol-excipient calibration mixtures

Candidate pharmaceutical excipients were chosen as examples of particular functions within a tablet formulation: Microcrystalline cellulose (binder, disintegrant), maize starch (diluent, binder) and magnesium stearate (lubricant). These were also selected as examples of excipients commonly used in paracetamol formulations [43, 44]. A series of different concentrations of paracetamol at 10.0%, 20.0%, 30.0%, 50.0%, 70.0% and 90.0% w/w in maize starch, MCC and magnesium stearate respectively were prepared mixing paracetamol and the excipient for 2 minutes. For the API/excipient calibration standard mixtures, a uniform total weight of 200mg was measured each time. These concentrations covered the different dosages in common OTC medicines. Spectral data, based on absorbance, was obtained using the OPUS software version 7.5 (Bruker Corporation, Germany). From these spectra, calibration data from the six different API concentrations, in the three separate excipients, were generated using the multivariate PLS Calibration Algorithm in the QUANT2 application on the OPUS software version 7.5. The regression equations obtained from the different analyses were used to determine the paracetamol content in the tablet samples. The final result was the mean of 5 separate runs per sample.

#### Processing of test tablet samples—Qualitative analyses

Individual tablet samples were removed from the blister pack and placed onto the sampling head of the ATR unit. Spectra were recorded where each spectrum was measured by averaging 20 scans over the range 4000-400cm^-1^ with spectral resolution 2cm^-1^. The estimated scan time for spectral acquisition was 25 seconds. This process was repeated 5 times to ensure replicate data was produced. These were recorded at several points on each side of the tablet in order to investigate data reproducibility and to assess if the presence of paracetamol, in OTC tablets, could be confirmed on the basis of this information.

#### Processing of test tablet samples—Quantitative analyses

Each tablet was weighed and then ground into fine powder using a mortar and pestle until a homogeneous mixture was obtained. Spectra of five individual powder samples per tablet were recorded. The data was processed using the multivariate analysis capability (PLS) of OPUS 7.5 QUANT2 software as detailed in the Reference paracetamol-excipient calibration mixtures section above. In any spectroscopic technique the measured signal is a function of the concentration of the analyte in the test matrix and in order to calculate the dose contained in a tablet it is necessary to accurately know the mass of the individual tablet analysed.

Measured levels of API were indicative of the percentage amount of API in tablet. The relationship between the results in % w/w and the actual dosage can be expressed as:

Actual Dosage of API in tablet (mg) = R x W

Where R = Concentration of API in % w/w and W = Total weight of the tablet (mg)

The levels of paracetamol in tablet medication obtained from the UK and several countries around the globe were then determined based on data obtained from the calibration mixtures.

#### UV–Vis analysis

Conventional solvent extraction method with UV analysis similar to the method cited by the British Pharmacopoeia was used to validate results already obtained for paracetamol tablets via ATR-FTIR. The protocol used by Behera et al [45] was adopted for this part of the study.

## Results and discussion

### Whole tablet qualitative analysis

**Fig 1:**
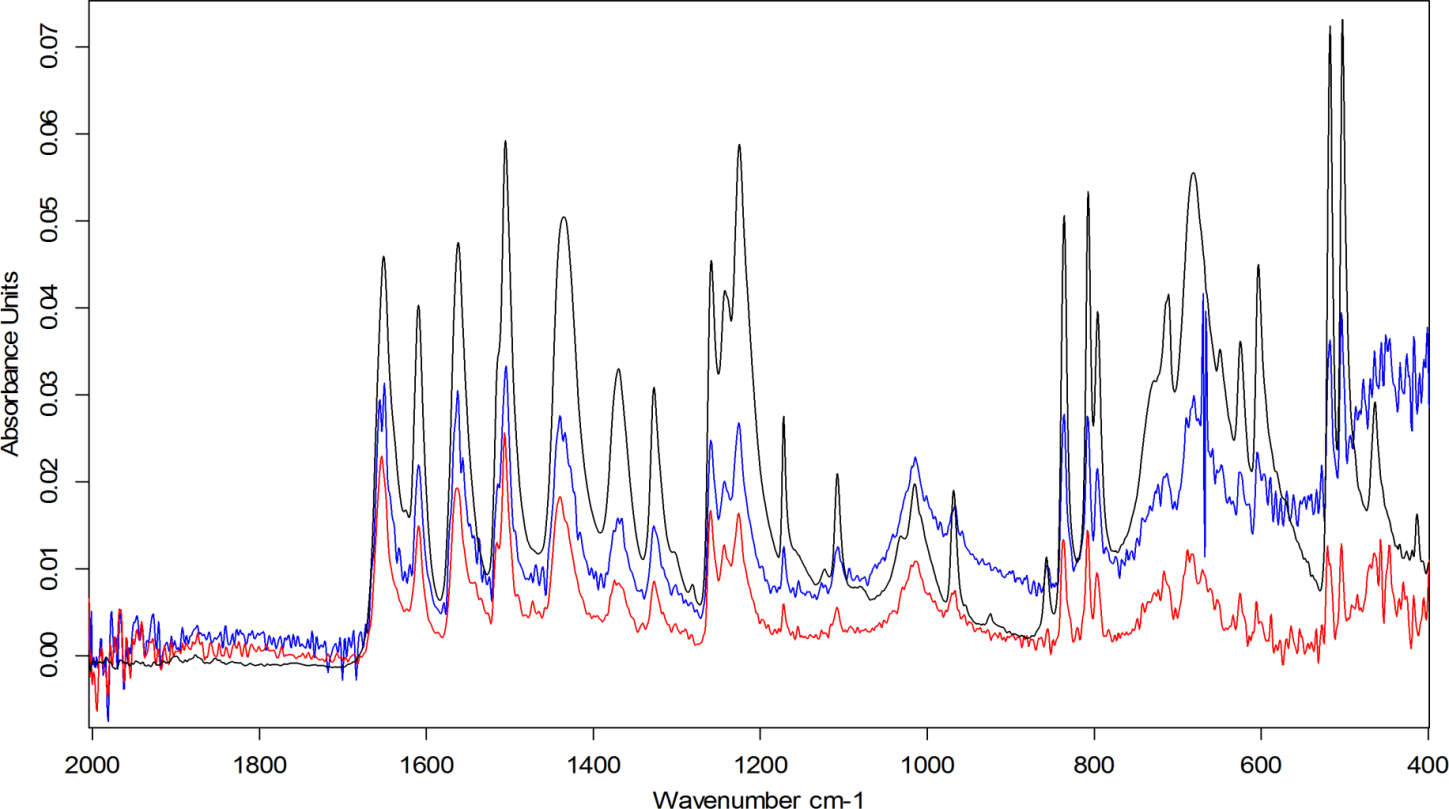
Paracetamol tablet crushed (Black), Whole tablet top (Blue), Whole tablet bottom (Red).

Data sufficiently reproducible to confirm the presence of paracetamol could be obtained from different analyses of a whole tablet placed on the sampling port of the ATR-FTIR instrument. This data in Fig 1 shows that peaks at the characteristic wavelengths of paracetamol are all present [23].

The different overall absorbance levels evident from the different sides of the same tablet (blue and red traces in Fig 1) result from different degrees of contact between the tablet surfaces and the ATR diamond surface. The degree of contact will depend on the surface characteristics of the tablet. A more intense and reproducible signal was obtained from the crushed powder samples shown in black in Fig 1.

### Spectral reproducibility

Good homogenisation was essential in order to obtain well defined, reproducible and quantifiable spectra. This was particularly important for the quantification of the API in tablet samples. Optimised grinding/mixing time was set at a minimum of 2 minutes per test tablet sample.

Overlays of replicate spectra of each reference material indicated that there were no detectable differences in either peak position or absorbance between replicate spectra of different samples of the same analyte. Initial studies showed some variation in peak intensities between replicate spectra but this was resolved with improved homogenisation of samples and proper reproducible covering of the sampling surface. Optimised sample preparation methods therefore gave reproducible spectral data across the range (4000–400 cm^-1^).

### Identification of API

In order to identify the API (paracetamol) in the presence of different excipients, a reference library containing spectra of reference material was created. Replicate spectra of the reference samples (paracetamol and excipients) were recorded and there was no detectable difference in absorbance bands and peak data between individual replicates of the same material provided instrumental conditions remained constant. Reproducible spectral data based on both absorbance bands and peak intensities were achieved after method optimisation.

#### Fingerprint and characteristic peaks for API identification

**Fig 2:**
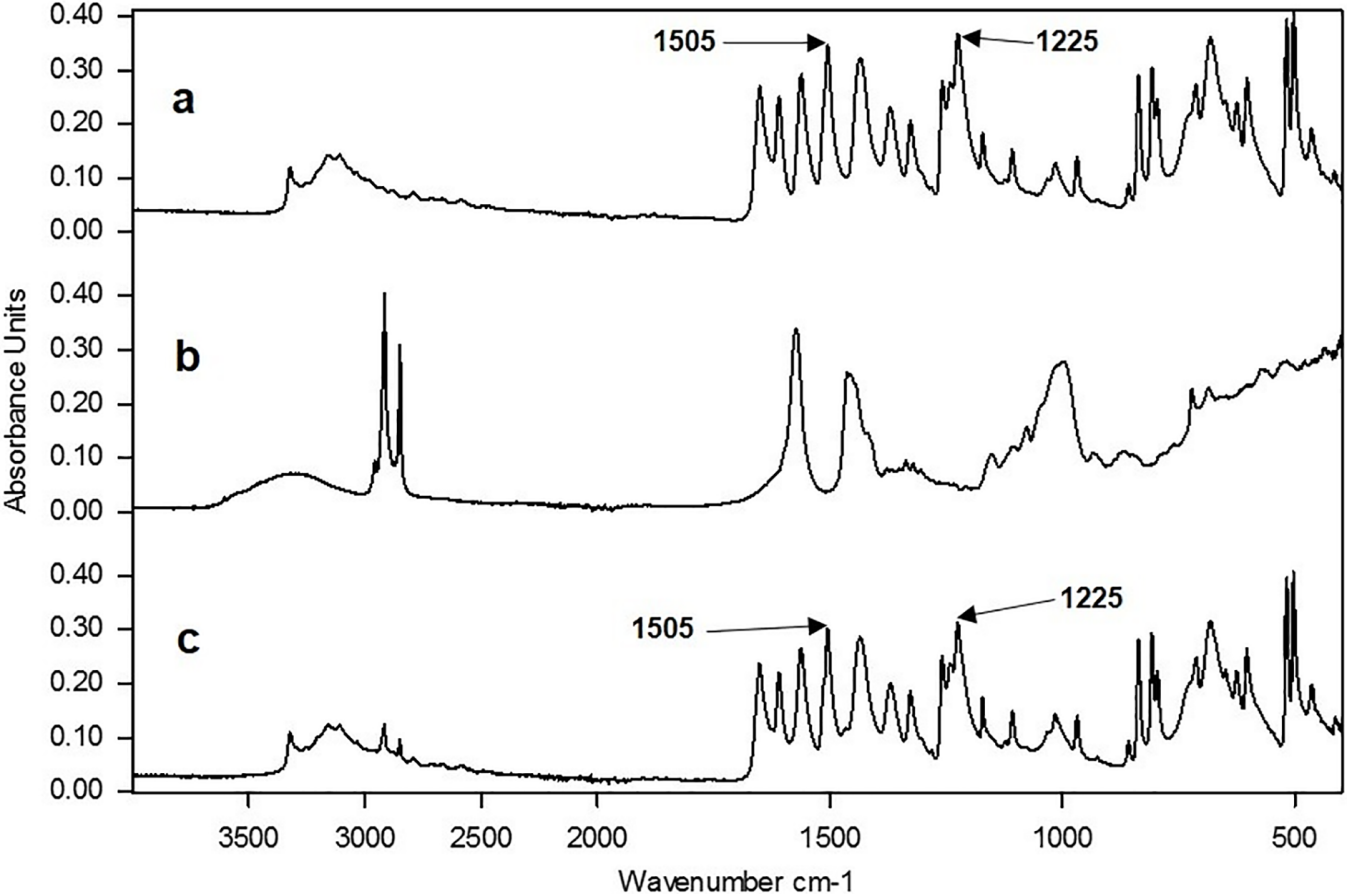
Comparison of three spectra: (a) pure paracetamol, (b) a mixture of three excipients (maize starch, magnesium stearate and microcrystalline cellulose), (c) a simulated paracetamol tablet mixture (80:20 paracetamol:excipients).

The reproducible reference library formed the basis for identification of OTC paracetamol tablet medications. Examples of reference spectra for paracetamol and an excipient mix are shown in Fig 2A and 2B respectively. Tablets are mixtures of the API and excipients. The spectrum of a model mixture of materials, for a paracetamol tablet containing 80% paracetamol, 10% maize starch, 5% microcrystalline cellulose and 5% magnesium stearate is shown in Fig 2C. The most noticeable change in Fig 2C is the reduction of the absorbance of the excipients bands between 2500 and 3000cm^-1^ as a result of the low concentration in the model tablet sample. This effect would also result in the reduction of all the bands below 1700cm^-1^ in the spectra from the excipients but this effect is masked by the API in Fig 2C.

Tablet spectra were then recorded and compared with those in the reference library and if the peaks in the fingerprint region (2000–400cm^-1^) matched, the presence of the API was confirmed. Aside from comparing the whole spectra, individual characteristic peaks were used to indicate the presence of a specified API in more complex tablet samples containing several excipients. This was achieved by selecting regions of the IR spectrum where there was little or no interference from the excipients. For example comparison of Fig 2A and 2C shows little difference in absorbance over the ranges 2000–1750cm^-1^, 1600 –1450cm^-1^ and 1300–1100cm^-1^.

**Fig 3:**
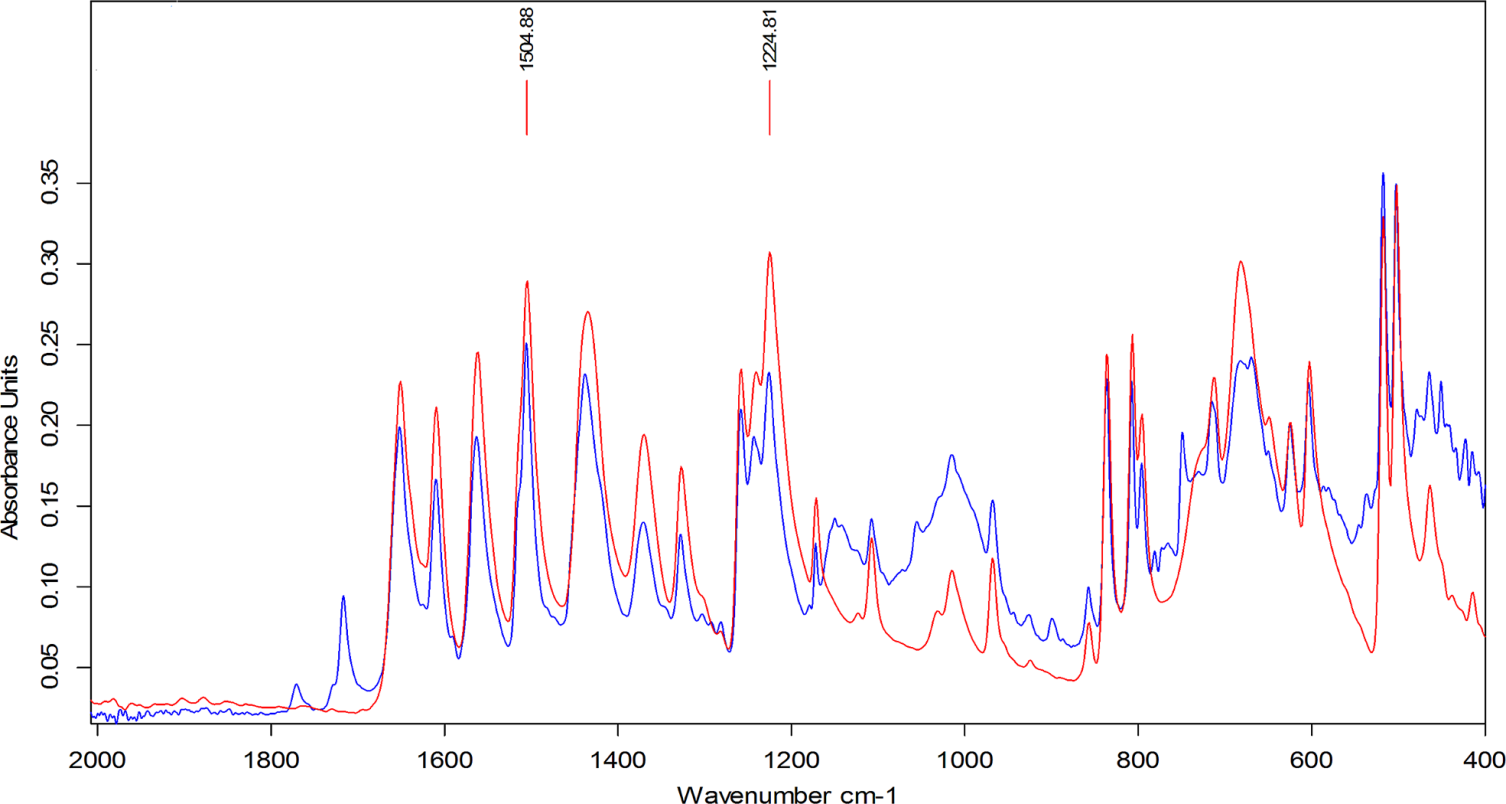
Overlay of ATR-FTIR spectra for identification: Pure paracetamol (red) and paracetamol tablet (blue).

This characteristic peak approach was compared with the mixture analysis application in the OPUS 7.5 software. This software application matches spectra by automatically combining up to four reference spectra available in the libraries. However, constituents of a mixture below 10% w/w could not be identified using this software. Fig 3 shows identification of paracetamol based on a comparison of spectra from pure paracetamol and a tablet formulation over the fingerprint region 2000–400cm^-1^. Identification of the presence of paracetamol was possible down to about 5% w/w of API in excipient using the two characteristic peaks cited in Fig 3.

**Fig 4:**
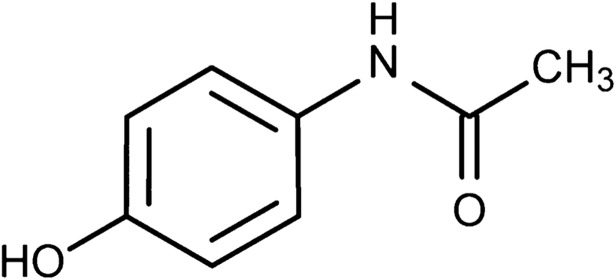
Molecular structure of paracetamol.

The structure of the paracetamol molecule is shown in Fig 4 and the peak at 1225cm^-1^ corresponds to the–OH in plane vibration and the peak at 1505cm^-1^ corresponds to the–CH_3_ vibration.

### Quantification of API using a multivariate PLS calibration model

During the preparation of the powder calibration mixtures magnesium stearate formed clumps and was difficult to mix uniformly. Both maize starch and MCC were easy to mix with paracetamol but the lower density of MCC may be an issue [46] when trying to ensure good contact on the sampling head.

Different approaches to the quantitative analysis of paracetamol were carried out by applying the OPUS 7.5 QUANT2 software application to the spectral data obtained from the calibration samples. The OPUS 7.5 QUANT2 application automatically employs a partial least square (PLS) regression approach to find the best correlation function between spectral and concentration data matrix.

The approaches trialed included the use of 3 individual excipients: MCC, maize starch and magnesium stearate with absorbance area measurements collected for:

- the ranges 1524–1493cm^-1^ and 1236–1210cm^-1^ corresponding to the 1505cm^-1^ and 1225cm^-1^ peaks,
- the range 1524 – 1210cm^-1^
- the complete spectral range 4000 – 400cm^-1^

**Fig 5:**
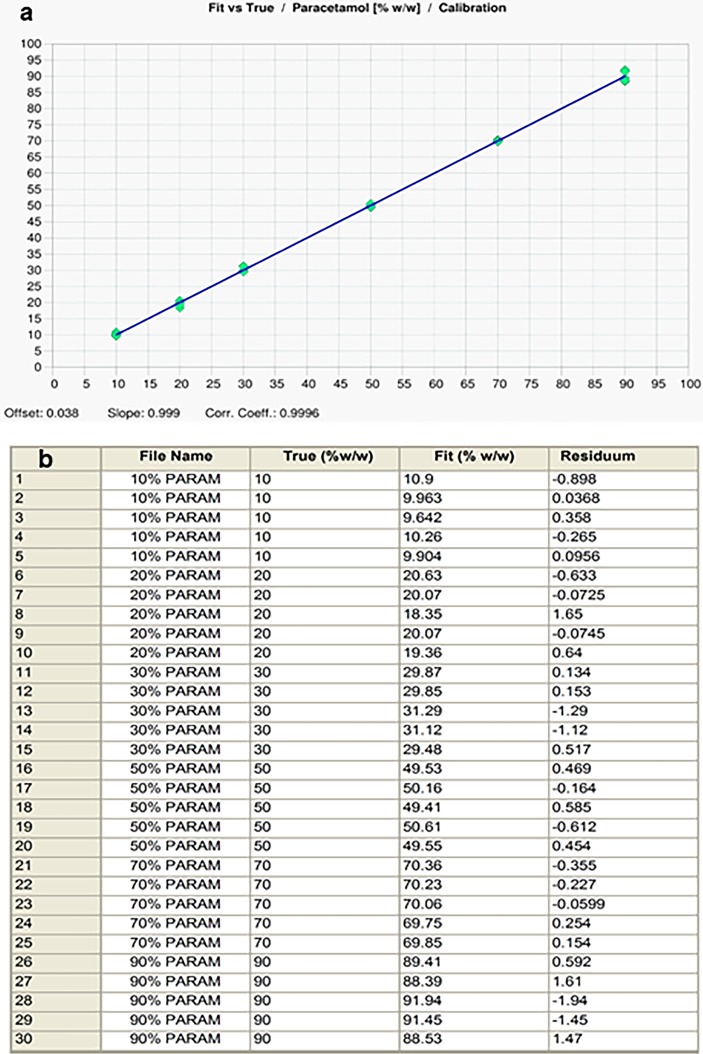
PLS calibration data for paracetamol: (a) PLS calibration plot for paracetamol over the range 1524-1493cm^-1^ (peak centred at 1505cm^-1^) from the Bruker QUANT2 software. Data is the mean of 5 replicates with maize starch as the excipient, (b) Comparison of measured (Fit) versus expected (True) amounts of paracetamol in calibration mixtures based on the range 1524 -1493cm^-1^.

Individual calibration curves for paracetamol in the different excipients, were plotted using the selected spectral ranges. Calibration graphs containing 10–90% paracetamol in the selected excipients were produced with R^2^ values between 0.96 and 0.99 for the different combinations. A representative example of the data produced is shown in Fig 5A and 5B for the range 1524-1493cm^-1^ (corresponding to the 1505cm^-1^ peak). Fig 5B is the comparison of the reference paracetamol concentrations (True) with measured concentrations (Fit) which showed close correlation using the PLS calibration model.

#### Methods validation

**Fig 6:**
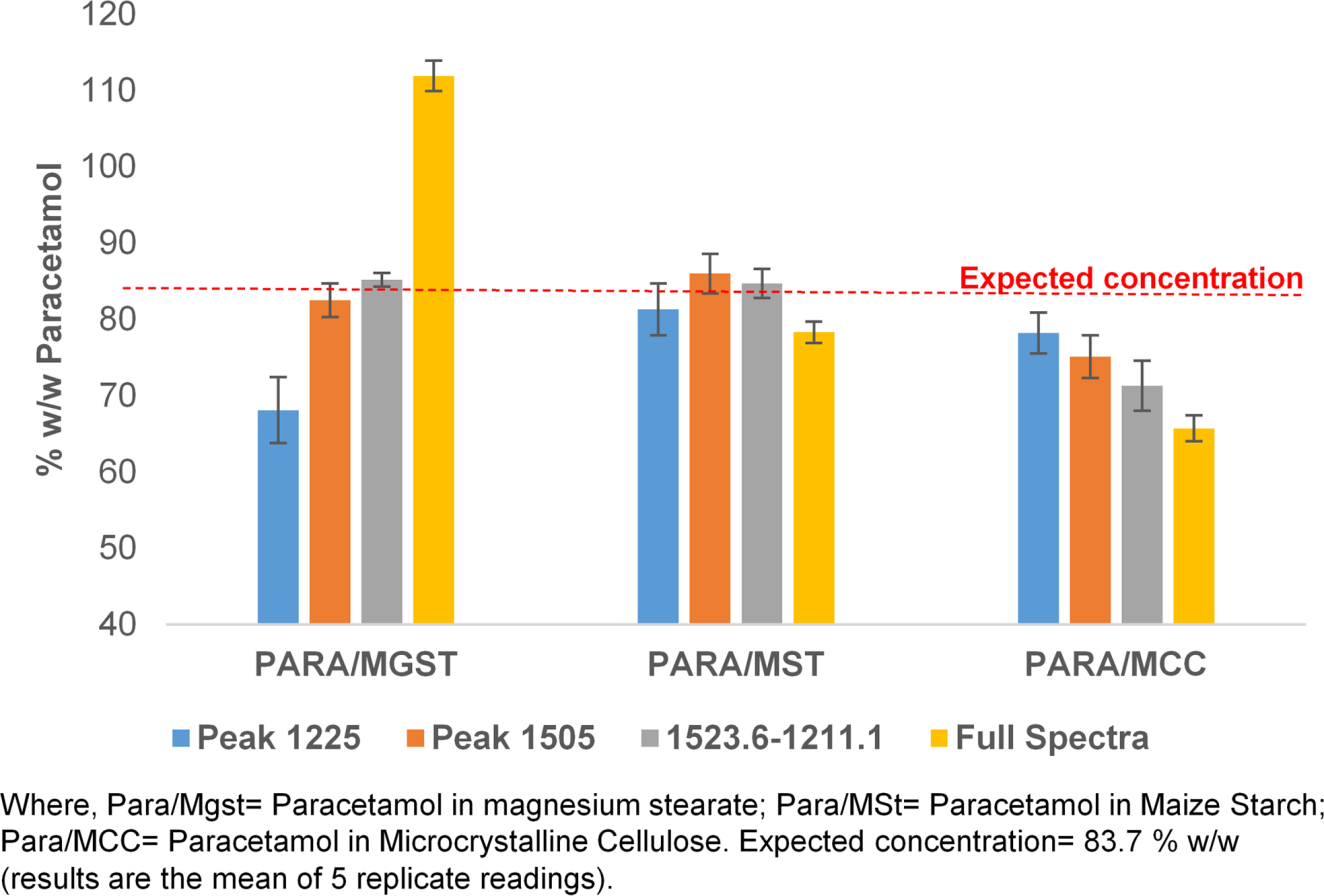
Chart showing measured amounts of paracetamol in tablets based on ATR-FTIR quantification methods using different absorbance bands and excipients.

The ability of the calibration approach based on the use of different excipients to correctly quantify the level of paracetamol in a tablet was assessed against a set of known typical OTC paracetamol tablets in which the paracetamol level had been measured by UV analysis. The tablets were assessed as containing 84% w/w paracetamol and the performance of the different ATR-FTIR approaches in assessing this value are shown in Fig 6. The results show that the measurements based on the maize starch calibration samples gave the most accurate and reproducible data set for all the spectral ranges covered. Data from the MCC based calibrants consistently under estimated the paracetamol levels whereas the magnesium stearate based data ranged from under estimation to significant over estimation of the API levels depending on the wavelength range chosen.

With the exception of the full spectrum mode the data obtained from the paracetamol in maize starch calibration samples accurately reflected the results obtained from the UV analysis of the test tablets. As a result of these experiments, the regression equation obtained from the calibration data of paracetamol in maize starch using the range of 1524 -1493cm^-1^ was used to determine the paracetamol content in tablet samples obtained from around the world.

#### Tablet sample analysis

Individual tablets from each of the separate samples were crushed and subjected to both the ATR-FTIR and UV analyses and the results for each sample set are recorded in Table 2. As can be seen most of UV and ATR-FTIR data agreed within ±10% of the expected dosage of the paracetamol tablets but some significant differences were identified. Whilst there was close agreement for many of the samples the ATR-FTIR approach gave high values versus UV for some samples from India and Cyprus. Low levels of API for samples from Pakistan were obtained from both UV and ATR measurements.

**Table 2:** Summary of the quantitative results for paracetamol tablet analysis from around the world (results are the mean of (n) number of samples).

Region	Country (Number of Tablets)	Tablets Analysed	UV Measured Content (mg)	ATR-FTIR Measured Content (mg)	Expected Amount (mg)
	**UK (n = 2)**	UK P1T1	532±4	514±15	500
		UK P1T2	479±3	505±15	
	**Cyprus (n = 2)**	Cyp P1T1	438±6	594±14	
		Cyp P1T2	442±6	591±5	
	**Switzerland (n = 2)**	Swz P1T1	523±6	510±10	
		Swz P1T2	508±5	505±9	
		Spn P1T1	529±7	514±15	
		Spn P1T2	515±6	497±18	
**Europe (n = 14)**	**Spain (n = 6)**	Spn P2T1	678±8	663±19	650
		Spn P2T2	648±5	683±10	
		Spn P3T1	1005±13	921±41	1000
		Spn P3T2	1008±10	980±20	
	**Belgium (n = 2)**	Bel P1T1	1031±11	1196±55	
		Bel P1T2	1090±11	1178±24	
**Asia & Middle East (n = 37)**	**India (n = 19)**	Ind P1T1	480±3	565±23	500
		Ind P1T2	545±5	558±21	
		Ind P2T1	521±5	462±11	
		Ind P2T2	539±3	469±16	
		Ind P3T1	464±4	493±16	
		Ind P3T2	499±4	500±7	
		Ind P3T3	528±4	493±9	
		Ind P4T1	478±3	489±8	
		Ind P4T2	550±5	481±25	
		Ind P5T1	504±3	533±20	
		Ind P5T2	539±4	542±16	
		Ind P5T3	485±3	509±25	
		Ind P6T1	487±7	545±6	
		Ind P6T2	463±6	540±8	
		Ind P7T1	502±7	530±23	
		Ind P7T2	442±6	529±15	
		Ind P8T1	365±3	464±12	325
		Ind P8T2	358±3	472±4	
		Ind P9T1	660±6	651±17	650
	**Pakistan (n = 2)**	Pak P1T1	449±6	373±14	500
		Pak P1T2	453±8	451±8	
	**Nepal (n = 8)**	Nep P1T1	501±5	532±13	
		Nep P1T2	498±6	526±18	
		Nep P2T1	497±7	501±17	
		Nep P2T2	475±7	461±17	
		Nep P3T1	450±4	535±13	
		Nep P3T2	494±3	555±7	
		Nep P4T1	517±6	499±24	
		Nep P4T2	552±5	536±11	
	**China (n = 4)**	Chn P1T1	541±4	494±21	
		Chn P1T2	495±3	500±9	
		Chn P2T1	488±3	516±11	
		Chn P2T2	548±4	546±6	
	**UAE (n = 4)**	UAE P1T1	525±7	527±8	
		UAE P1T2	513±6	541±17	
		UAE P2T1	484±5	499±12	
		UAE P2T2	500±6	520±16	
**Africa and Caribbean Islands (n = 14)**	**Rwanda (n = 6)**	Rwa P1T1	543±4	508±9	500
		Rwa P2T1	476±3	525±8	
		Rwa P3T1	511±4	486±14	
		Rwa P3T2	581±4	536±11	
		Rwa P4T1	503±3	533±10	
		Rwa P4T2	519±4	519±14	
	**Ghana (n = 4)**	Gha P1T1	476±5	496±20	
		Gha P1T2	504±5	510±11	
		Gha P2T1	508±6	530±5	
		Gha P2T2	479±6	490±13	
	**Jamaica (n = 4)**	Jam P1T1	510±5	481±11	
		Jam P1T2	515±6	498±15	
		Jam P2T1	533±6	509±10	
		Jam P2T2	511±7	517±15	

**Fig 7:**
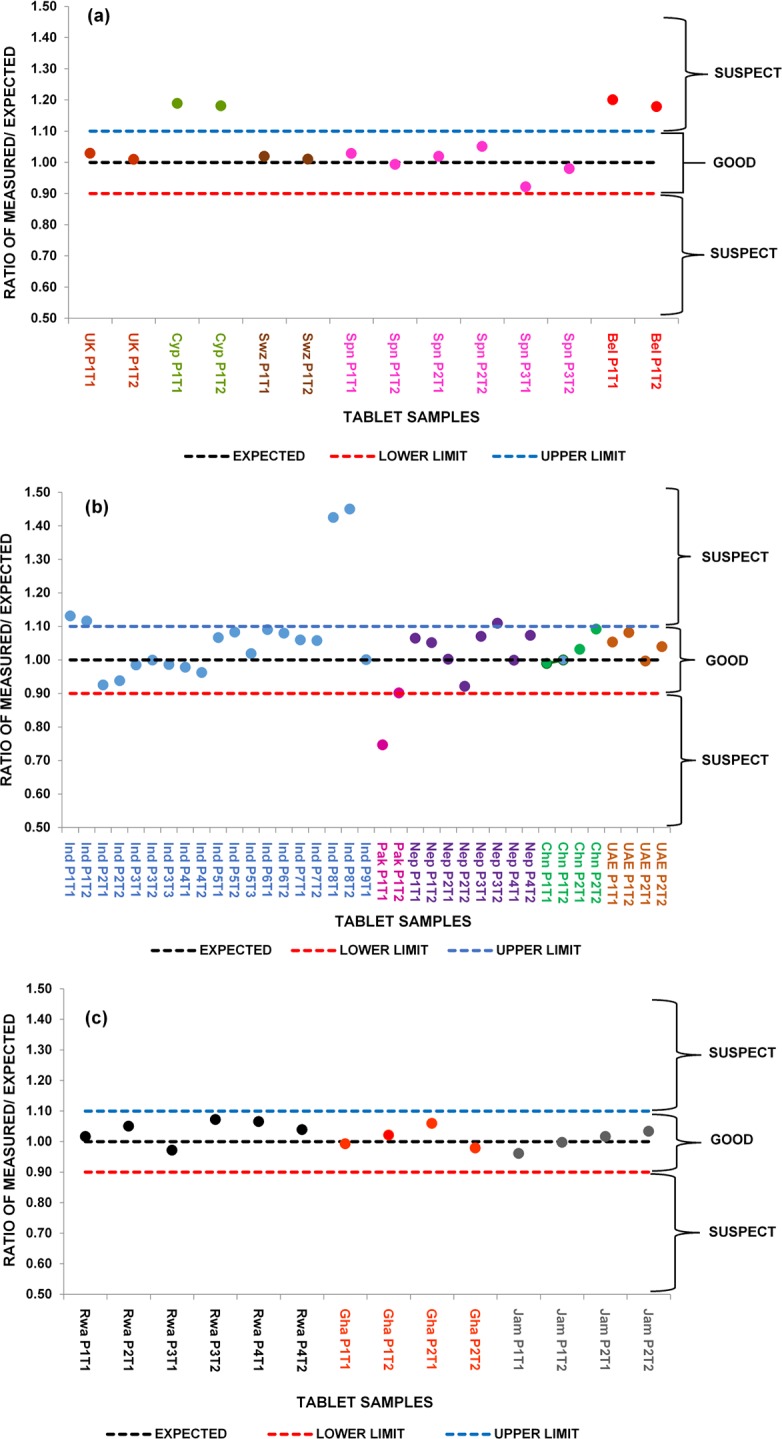
Ratio of measured to expected amounts of Paracetamol in tablet samples from around the world based on calibration for peak over the range 1524-1493cm^-1^ (centred at 1505cm^-1^): (a) Ratio of measured to expected amounts of Paracetamol in 14 samples of tablets from around Europe, (b) Ratio of measured to expected amounts of Paracetamol in 37 samples of tablets from Asia and Middle East, (c) Ratio of measured to expected amounts of Paracetamol in 14 samples of tablets from around Africa and the Caribbean Islands.

The ATR-FTIR data can be more meaningfully displayed as a plot of the ratio of expected to measured paracetamol levels versus sample origin as shown in Fig 7A–7C. These diagrams clearly show that whilst the majority of tablet samples were within the acceptable limits, four tablet samples merited further investigation.

The first of these, the Belgian tablet samples, gave similarly high results for both the UV and the ATR-FTIR tests and would be allowed to pass a qualitative screening test (Fig 7A). The tablet samples from Cyprus would also fail a screening test with too little paracetamol, based on the UV data and too much apparent paracetamol based on the ATR-FTIR (Fig 7A). Both results do, however, have the same overall affect, this set of tablets would require further analysis. It was noted that the Cyprus paracetamol tablets contained glycerol as an excipient and in this respect were different to the other tablets investigated. Glycerol is not a common pharmaceutical excipient and its FTIR spectrum shows that it has significant absorbance in the range 1524 -1493cm^-1^ [47] where the measurements were taken in this study and could therefore be the reason for the high ATR-FTIR responses. One advantage of the ATR-FTIR approach taken here, recording reference and sample spectra over the range 4000-400cm^-1^ means that the Spectrum Search capabilities of the OPUS 7.5 software can be used. This approach should allow identification of the compound/s contributing to the anomalously high peak area centred on 1505cm^-1^. This will be the subject of a future publication.

Two of the Indian tablet samples (ratio 1.44) tested high on both UV and ATR-FTIR but the ATR-FTIR signal suggested the presence of other material in the fingerprint region and would therefore fail a screening test (Fig 7B). Further investigation of the reason for failure would be required. For the Pakistan samples (ratio 0.75) both the UV and ATR-FTIR analyses showed clear evidence of insufficient levels of paracetamol (Fig 7B).

The developed qualitative and quantitative ATR-FTIR methods have a number of advantages as they not only identify the presence or absence of the API (paracetamol) but also indicate how much of the API could be in the tablet in a short time. They also reduce exposure to toxic chemicals used in solvent extraction of the API(s) for analysis using conventional pharmacopoeia approved methods. The ATR-FTIR instrument used in this study is small and compact and therefore its portability makes it valuable for in-field analysis such as quality control by pharmaceutical companies and post marketing surveillance by regulatory bodies. Furthermore, for applications to medicines other than paracetamol the potential for the identification of falsified and substandard medicines with incorrect amounts of API will also reduce the public health risk posed by these medications such as antimicrobial resistance and ultimately therapeutic failure. The dangers of under dosing or exceeding the allowed limits for API(s) in medication especially those with a narrow therapeutic range will also be reduced. Economically, funds spent on these medications which are toxic or have no therapeutic effect will be reduced.

## Conclusion

The overall result of this study, the identification of 7 suspect paracetamol tablet samples or 12%, is broadly in line with the WHO estimates for the general level of falsified or substandard medicines worldwide. This level of suspect tablet formulations reflects the eclectic source of the samples collected in this work.

This study demonstrates that the simple ATR-FTIR approach employed has the capacity to rapidly identify and also quantify paracetamol in the presence of excipients. The whole process of crushing, identifying and quantifying a tablet would take about 5 minutes per tablet sample after the method has been optimised. The multivariate PLS calibration model used in this study is an automated process further speeding up the time for data processing. This automated method further reduces variation in data due to errors in manual integration of characteristic peaks for paracetamol identified. However, this is not meant to replace the more established and highly sensitive conventional solvent extraction methods but to serve as an alternative to the more expensive Raman systems as an in-field technique for quick screening of medicines. It is also a green technique as the elimination of solvent extraction of APIs reduces the amounts of toxic chemicals used thus, reducing chemical waste. Furthermore, the technique will enable quick withdrawal of substandard and falsified medicines from the market thereby reducing the threat to public health. The ATR-FTIR approach reduces testing of tablets to three simple steps; crush tablet, measure powder and read results whereas the conventional UV spectrophotometric analysis requires solvent extraction, dilution and filtration prior to analysis and generation of results. It is also relatively inexpensive and easy to use compared to the pharmacopoeia approved techniques so can possibly be used in LMICs where facilities are not readily available. This approach employed in the identification and quantification of paracetamol could potentially be applied in the analysis of other APIs in tablet dosage forms.
